# Real-world occurrence, therapy, and outcome of patients with class 2 or 3 BRAF compared with class 1 BRAF-mutated cancers

**DOI:** 10.1016/j.esmorw.2024.100075

**Published:** 2024-09-25

**Authors:** S. Pradervand, N. Freundler, B. Gosztonyi, L. Roncoroni, R. Achermann, T. Schwenk, G. de Fraipont, J. Garessus, S. Haefliger, A.B. Leichtle, M.K. Kiessling, T. Mueller-Focke, F.S. Krebs, V. Zoete, P. Tsantoulis, O. Michielin, C. Britschgi, A. Wicki

**Affiliations:** 1 Centre Hospitalier Universitaire Vaudois – CHUV, Department of Oncology, Lausanne; 2 Department of Medical Oncology and Hematology, University Hospital Zurich, University of Zurich; 3 Department of Medical Informatics, Universitätsspital Basel – USB, Basel; 4 Oncology, Hematology and Transfusion Medicine, Kantonsspital Aarau, Aarau; 5 Geneva University Hospitals – HUG, Geneva; 6 Department of Medical Oncology, Inselspital, Bern University Hospital, University of Bern; 7 Department of Clinical Chemistry, Inselspital – Bern University Hospital and Center for Artificial Intelligence, University of Bern, Bern; 8 Computer-Aided Molecular Engineering Group, Department of Oncology UNIL-CHUV, Ludwig Institute for Cancer Research Lausanne, Lausanne; 9 Molecular Modelling Group, Swiss Institute of Bioinformatics, Lausanne; 10 Swiss Group for Clinical Cancer Research (SAKK), Bern, Switzerland

**Keywords:** BRAF mutations, precision oncology, personalized treatment, Swiss Personalized Health Network (SPHN)

## Abstract

**Background:**

*BRAF* V600 mutations are the epitome of targeted therapy. However, not much is known about non-V600 mutations. Using the new data infrastructure of the Swiss Personalized Oncology project of the Swiss Personalized Health Network (SPHN), we evaluated the fate of patients with cancer with non-V600 *BRAF* mutations in comparison to patients with class 1 mutations.

**Patients and methods:**

In this retrospective observational multicenter study, we have assembled a cohort of 392 patients with class 1 and 154 patients with nonclass 1 *BRAF* mutations (76 colorectal cancers, 96 lung cancers, 297 melanomas, and 77 other cancers). We carried out outcome analyses between mutational classes and therapeutic subgroups.

**Results:**

Overall survival (OS) did not differ significantly between patients with class 1 and nonclass 1 mutations. Upon treatment with BRAF/MEK inhibitors, patients with class 1 mutant melanoma showed numerically longer progression-free survival (PFS; 217 days) than patients with nonclass 1 mutant disease (73 days). Overall, in patients with class 2 or 3 mutations, BRAF and MEK inhibitors showed no benefit over other systemic therapies. However, specific class 2 mutations such as K601E may confer sensitivity to BRAF/MEK inhibitors, with two out of five patients achieving a PFS >400 days.

**Conclusions:**

The diversity of *BRAF* mutations presents significant treatment challenges. Despite similar OS, nonclass 1 mutant tumors showed a trend toward lower PFS with BRAF/MEK blockade. Selected class 2 mutations may confer sensitivity to BRAF/MEK inhibitors. This highlights the rationale for a mutation, rather than class-specific, clinical approach against nonclass 1 BRAF-mutant tumors.

## Highlights

• In metastatic disease, OS did not differ between patients with class 1 and nonclass 1 BRAF mutations.
• PFS was numerically lower with BRAF/MEK inhibitors in nonclass 1 compared with patients with class 1 mutant melanoma.
• We observed two patients with a class 2 K601E mutation responding for >400 days to combined BRAF/MEK inhibition.
• Our data suggest that treatment for BRAF-mutant disease could benefit from a mutation rather than a class-specific approach.

## Introduction

Oncogenic *BRAF* alterations occur in a broad range of cancers.^1^ With increasing numbers of sequenced tumor genomes, it became evident that *BRAF* mutations fall into three distinct classes.^2^ First-generation BRAF inhibitors (BRAFis; vemurafenib, dabrafenib, and encorafenib) target only class 1 mutations, particularly the V600 hotspot mutation. In melanoma, the focus of interest for early BRAF p.V600 inhibitor development, these drugs have shown impressive overall survival (OS) benefits.^3^ As resistance to BRAFis can develop rapidly, and all currently available inhibitors induce paradoxical CRAF activation,^4^ which can lead to secondary squamous cell carcinomas, BRAFis have been combined with MEK inhibitors (MEKis).^5^, ^6^, ^7^

BRAFis alone and in combination with MEKis have been tested in a tissue-agnostic approach in nonmelanoma cancers. In these tumors, the median frequency of *BRAF* mutations is ∼8%.^2^ Response rates are between 38% and 89%.^8^, ^9^, ^10^, ^11^ Based on this, the combination of dabrafenib and trametinib received Food and Drug Administration (FDA) approval in June 2022 for all *BRAF* p.V600E-mutated cancers, regardless of the tissue of origin, with the explicit exception of colorectal cancer.^12^ In *BRAF*-mutant colorectal cancer, particularly low response rates to BRAF and MEK blockade have been observed.^13^ This is due to the rapid feedback activation of epidermal growth factor receptor (EGFR), which allows for continued growth despite BRAF inhibition. However, the combination of anti-EGFR antibodies with BRAFis has been more successful in this entity.^14^^,^^15^

While the clinical utility of class 1 *BRAF* mutations is well established, there is limited evidence about class 2 and 3 *BRAF* mutations and their response to BRAF or MAPK pathway inhibitors. Class 2 mutations, for example, in codons 597 and 601, activate the BRAF kinase domain. Case reports and small series suggest moderate clinical activity of downstream MEK inhibition, particularly in patients with melanoma.^16^ However, no robust trial has yet addressed this aspect.

Class 3 mutations do not increase kinase activity but may facilitate upstream signaling through *RAS* mutations or receptor tyrosine kinase (RTK) binding, making downstream MEK inhibition less promising. It is recognized that class 2 and 3 *BRAF* mutations are challenging to target with the currently approved agents against class 1 *BRAF* mutations and that MEKis (cobimetinib, trametinib, and binimetinib) may have minimal activity in class 2, and virtually no activity in class 3 mutant disease.^17^

The federal Swiss Personalized Health Network (SPHN) harmonizes and shares clinical data across Switzerland. The Swiss Personalized Oncology (SPO) project, an SPHN subproject, structures and unites oncology data between the five Swiss university hospitals using specified semantic elements (the SPHN ‘concepts’), a flexible data exchange format, the BioMedIT network for secure data processing, and a uniform set of 110 oncology-related parameters (SPO minimal data set).^18^, ^19^, ^20^

Using the SPO network, we have previously shown that machine learning approaches can predict the class of cancer-associated *BRAF* mutations.^21^ In this clinical pilot project of the SPO network, we investigated the frequency and class of *BRAF* mutations across all cancer entities in the participating institutions and assessed their correlation with baseline characteristics, therapies, response rates, and survival.

## Patients and methods

### Cohort assembly

**Figure 1:**
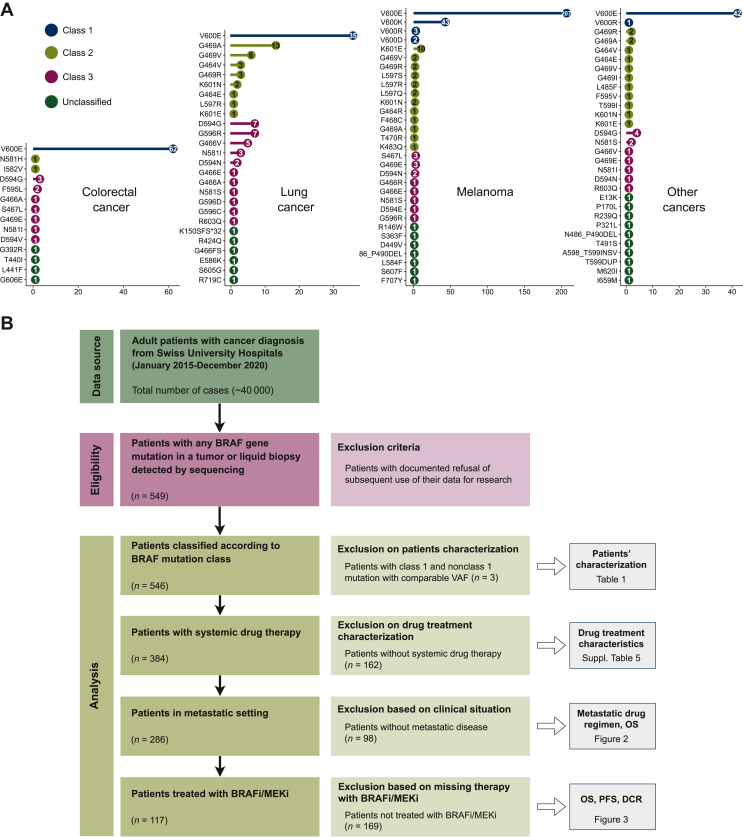
(A) Cleveland dot plot with the number of occurrences of the different *BRAF* mutations stratified by cancer types (colorectal cancer, lung cancer, melanoma, and other cancers). Mutations are colored by class. (B) Study flow diagram. BRAFi, BRAF inhibitor; DCR, disease control rate; MEKi, MEK inhibitor; OS, overall survival; PFS, progression-free survival.

For this retrospective, observational, multicenter study (ethics approval: BASEC ID 2020-00347), patients who were tested for a *BRAF* mutation between 1 January 2015 and 31 December 2020 (Figure 1) at the five university hospitals in Switzerland were included. Patients who refused general consent for research were not eligible.

### Data collection and processing

Data were collected using the SPHN reference dataset (version 2021.1) and our project-specific extensions.^20^ The dataset was represented with a Resource Description Framework (RDF) schema, encrypted, and securely transferred to the BioMedIT network.^18^^,^^19^ To ensure semantic harmonization, a set of semantic rules written in shapes constraint language (SHACL) was defined and distributed together with the ontology. To prevent erroneous content and ensure interoperability, we integrated a set of queries in a data validation pipeline. Integrity checks were carried out to investigate possible missing data points and inconsistencies in patient timelines.

Metastatic treatment lines were reconstituted according to progression dates. Drugs were grouped into regimens according to their administration dates and manually corrected by medical experts.

### Genomic profiling

At each site, liquid and solid biopsies were profiled using various routine clinical, in-house validated technologies. Next-generation sequencing panels were carried out by both hybrid-capture and amplicon-based methods, such as FoundationOne, Oncomine, and Ion AmpliSeq, and sequenced on Illumina (NextSeq, MiSeq), and Ion Torrent (Ion Proton, Ion PGM). Sanger sequencing, pyrosequencing, Droplet Digital PCR, and a cell-free DNA assay were also used (Supplementary Material, available at https://doi.org/10.1016/j.esmorw.2024.100075).

### *BRAF* mutations

*BRAF* indels and single-nucleotide variants were classified according to Yao et al.,^22^ Dankner et al.,^23^ and Lin et al.^24^ Mutations not referenced in these reports were classified into class 2 or 3 using the model developed by Krebs et al.^21^ (Supplementary Table S1, available at https://doi.org/10.1016/j.esmorw.2024.100075).

### Statistical method

Unless otherwise stated, all statistical computations were carried out with the R statistical tool (version 4.3.3 2024-02-29; R Foundation, Vienna, Austria). All *P* values are two-sided, with an alpha level set at 0.05. As the numbers were not large, we used the exact Fisher’s test for contingency tables and the binomial test for testing single empirical proportions with a value of 1/2 as the null hypothesis and the multinomial test for multiple proportions with a value of 1/*n* as the null hypothesis, where *n* is the number of categories. The 95% confidence interval (CI) associated with the binomial test was computed with the Clopper–Pearson method and the simultaneous 95% CIs associated with the multinomial test were estimated with the Goodman method.

### Survival analysis

Kaplan–Meier estimates and Cox proportional hazard ratios were computed in R using packages survival (3.2.7) and survminer (0.4.9) with default parameters. The survival curves were built using Kaplan–Meier’s estimator. Hazard ratios were computed through Cox’s regression model. The *P* value of the Kaplan–Meier estimator was computed from the log-rank test statistics, and the hazard ratio *P* value was derived from the *Z*-score of the parameter estimate. The proportional hazards assumption for Cox regressions was checked by assessing the noncorrelation between time and scaled Schoenfeld’s residuals and by graphical evaluation (data not shown). We used the Kaplan–Meier potential follow-up to estimate the median follow-up of each group.

For progression-free survival (PFS), if the patient is not deceased and has only assessments reporting nonprogressive disease, the last date of follow-up and treatment assessment was used as a censoring event. Patients for whom neither the event date nor the censoring date could be established were excluded.

Disease progression dates were transcribed from medical records by oncologists. The rate of nonprogression or disease control rate (DCR) was calculated as defined.^25^ In the presence of a censoring event occurring before the specified DCR time point, we removed the patients from the count related to that time point.

## Ethics statement

This study (Protocol No. 2020-00347) was approved by the Northwest and Central Swiss Ethics Committee (EKNZ) and ratified by the local ethics committees (CCER, CER-VD, Kantonale Ethikkommission Bern, Kantonale Ethikkommission Zürich).

## Results

### Data collection through the Swiss Personalized Oncology network

We inventoried adult oncology patients at the five Swiss university hospitals for whom a *BRAF* gene mutation was detected by sequencing between 1 January 2015 and 31 December 2020. We identified 558 *BRAF* mutations in 549 patients (Figure 1A and B) with 395 class 1, 72 class 2, and 64 class 3 mutations; 27 *BRAF* mutations could not be classified. Their amino acid sequences are known, but their influence on BRAF catalytic activity is neither known nor predictable with sufficient confidence.

### Characteristics of patients with cancer with *BRAF* mutations

Patients were categorized according to the class of their *BRAF* mutation. Three patients with a class 1 mutation and a concomitant nonclass 1 mutation with a comparable variant allele frequency^26^ were excluded from further analysis, resulting in 392 class 1 and 154 nonclass 1 patients (Supplementary Table S2, available at https://doi.org/10.1016/j.esmorw.2024.100075). Age, sex, cancer type, stage, and treatment type were compared for the different mutation classes (Table 1, Supplementary Tables S3–S5, available at https://doi.org/10.1016/j.esmorw.2024.100075).

**Table 1:** Characteristics of patients classified according to their BRAF mutation (546 patients)

Characteristics	Class 1	Class 2	Class 3	Unclassified
	392	70	62	22
**Age at diagnosis, years**				
Median (Q1-Q3)	63 (51-73)	70 (63-76)	71 (63-77)	53 (40-66)
**Administrative gender, *n* (%)**				
Female	177 (45.2)	30 (42.8)	28 (45.2)	8 (36.4)
Male	215 (54.8)	40 (57.1)	34 (54.8)	14 (63.6)
**Diagnosis, *n* (%)**				
Colorectal cancer	61 (15.6)	2 (2.9)	10 (16.1)	3 (13.6)
Non-small-cell lung cancer/small-cell lung cancer	33 (8.4)	30 (42.9)	28 (45.1)	5 (22.7)
Melanoma	255 (65.1)	25 (35.7)	13 (21.0)	4 (18.2)
Other	43 (11.0)	13 (18.6)	11 (17.7)	10 (45.5)
**Stage, *n* (%)**				
I	18 (5.0)	6 (9.0)	8 (14.3)	0 (0)
II	17 (4.7)	5 (7.6)	2 (3.6)	0 (0)
III	93 (25.6)	9 (13.6)	7 (12.5)	1 (5.3)
IV	235 (64.7)	46 (69.7)	39 (69.6)	18 (94.7)

For each *BRAF* mutation class, relative percentages are indicated. The stage at BRAF mutation diagnosis is reported. Cancer stage information is missing for 42 patients.

Our cohort includes a large number of patients with melanoma with class 1 mutations (255/546). The ratio of *BRAF* class 1 to nonclass 1 mutations was higher in melanoma (255 versus 42, 85.9%) than in other cancers with *BRAF* mutations (137 versus 112, 55.0%), with an odds ratio of 4.95 (95% CI 3.23-7.68, *P* < 0.001, Fisher’s exact test). In melanoma, as reported in other studies, we also observed a gender disparity when looking at all patients with a *BRAF* mutation (60.3% male versus 39.7% female; *P* < 0.001; binomial test; Supplementary Table S3, available at https://doi.org/10.1016/j.esmorw.2024.100075).^27^ In other cancers, the sex ratio was evenly distributed. The male-to-female ratios within the different BRAF mutation classes do not differ significantly from each other (Supplementary Table S4, available at https://doi.org/10.1016/j.esmorw.2024.100075).

Regarding the cancer stage, the average proportion of stage IV was 67.1%. This did not vary between the first three classes: 64.7%, 69.7%, and 69.6%, but we observed a deviation in the 27 unclassified mutations with 94.7% stage IV patients (*P* = 0.0324, Fisher’s exact test). This is probably because earlier-stage disease is more likely to be tested with hotspot gene panel assays rather than with comprehensive panels, which may not capture rare, lesser-known mutations. We lack information about the stage for 42 patients (29 of class 1, 4 of class 2, 6 of class 3, and 3 unclassified).

As reported in previous studies,^25^ the relative proportions of mutation classes varied by cancer type (Table 1). Patients with non-small-cell lung cancer/small-cell lung cancer showed an even distribution of the mutations among the three classes: 36.3% class 1, 33.0% class 2, and 30.8% class 3 (*P* = 0.827; multinomial test; 95% CIs 0.253-0.488, 0.225-0.455, and 0.206-0.432 for classes 1, 2, and 3, respectively]. A preponderance of class 1 mutations is observed in melanoma (87.0% class 1, 8.53% class 2, and 4.44% class 3; *P* < 0.001; 95% CI 0.816-0.910, 0.0538-0.133, and 0.0233-0.0829, respectively) and colorectal cancer (83.6% class 1, 2.74% class 2, 13.7 % class 3; *P* < 0.001; 95% CI 0.708-0.914, 0.00591-0.118, and 0.0670-0.260). In colorectal cancer, we also observed a higher proportion of class 3 mutations than class 2 mutations (*P* = 0.039; binomial test; 95% CI 0.516-0.979). In melanoma, the proportion of class 2 mutations is higher than class 3, but not significantly different (*P* = 0.073; binomial test; 95% CI 0.486-0.804).

### Drug treatment characteristics

**Figure 2:**
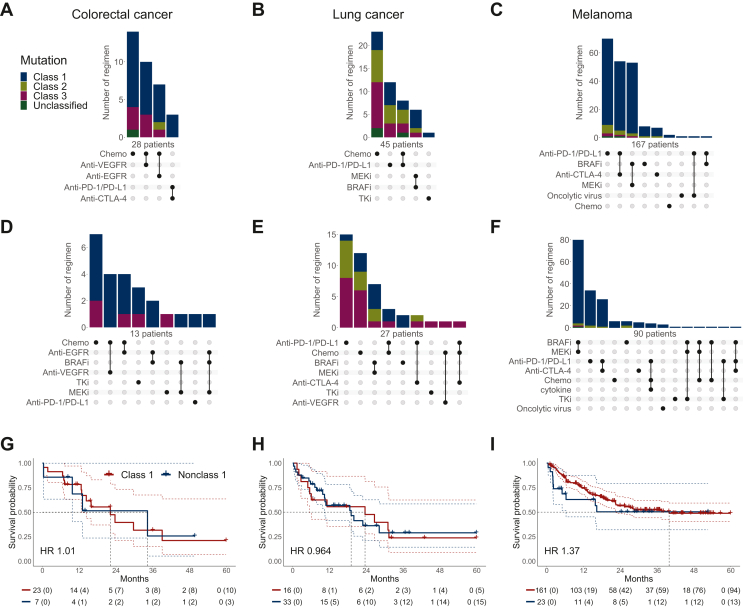
Patients with *BRAF*-mutated metastatic disease, including those with (A, D, G) colorectal cancer, (B, E, H) lung cancer, and (C, F, I) melanoma receiving drug treatment. UpSet plot of drug treatment regimens given to patients as (A–C) first metastatic line and (D–F) second and further metastatic lines. Numbers of regimens are indicated, grouped by *BRAF* mutation classes. All chemotherapies are grouped together (chemo). (G–I) Kaplan–Meier estimators of overall survival, stratified by class of *BRAF* gene mutation. The number of patients at risk at each time point is indicated below the plot, together with the cumulative number of censoring events in parentheses. Survival starts from the first application of therapy for metastatic disease. The censorship, if any, occurs in the last follow-up event observed when the patient is alive. Colored dashed lines represent the upper and lower boundary of the pointwise 95% confidence interval. The median survival is shown with black dashed lines. The number of events and median survival are indicated in Supplementary Table S6, available at https://doi.org/10.1016/j.esmorw.2024.100075. BRAFi, BRAF inhibitor; CTLA-4, cytotoxic T-lymphocyte associated protein 4; EGFR, endothelial growth factor receptor; HR, hazard ratio; MEKi, MEK inhibitor; PD-1, programmed cell death protein 1; PD-L1, programmed death-ligand 1; TKi, tyrosine kinase inhibitor; VEGFR, vascular endothelial growth factor receptor.

Of 549 patients with *BRAF* mutations, 384 received systemic antineoplastic therapy, including 286 in the metastatic setting (Figure 1B). The metastatic treatment lines of each entity, stratified by first and subsequent lines, are detailed in Figure 2.

Among the 28 patients with colorectal cancer who received systemic therapy for metastatic disease, consisting mainly of class 1 and class 3 mutant disease, chemotherapy was the most common first (14/28, 50%) and subsequent treatment line (7/13, 54%), followed by chemotherapy combinations with targeted antivascular endothelial growth factor receptor and anti-EGFR agents across all lines (Figure 2A and D). Of the five patients treated with immunotherapy in any setting (Supplementary Table S5, available at https://doi.org/10.1016/j.esmorw.2024.100075), all had a class 1 mutation and four with co-occurrence of mismatch repair deficiency.

In patients with *BRAF* class 1 metastatic lung cancer, BRAFis/MEKis were more prevalent in subsequent (6/9, 67%) than in first-line treatments (4/15, 27%; Figure 2B and E). Of all patients with lung cancer with class 2 and 3 mutations, 7% (2/28) received BRAF and/or MEK inhibition in first, and 17% (3/18) in subsequent lines for metastatic disease (Figure 2B and E).

In melanoma, among all 174 patients with metastati disease across all *BRAF* classes, BRAF and/or MEK inhibition (55/167, 33%) was the second most common first-line treatment after immunotherapy (Figure 2C). In subsequent treatment lines, class 1 mutant disease was predominantly treated with BRAFis/MEKis (63/83, 80%), while class 2 mutant disease was similarly often treated with either immunotherapy (3/5, 60%) or BRAF/MEK targeted therapy (3/5, 60%; Figure 2F).

Of the 39 patients with other cancer types in any treatment setting (Supplementary Table S5, available at https://doi.org/10.1016/j.esmorw.2024.100075), chemotherapy was the most common treatment (82%), followed by targeted treatments (41%) and immunotherapies (33%).

Regarding any treatment setting, the rate of targeted treatments (BRAFi/MEKi and other targeted agents) in tumor-agnostic class 2 *BRAF*-mutant disease (10/41, 24%) was lower than in class 1 *BRAF*-mutant disease (166/289, 57%) but comparable to class 3 *BRAF*-mutant disease (13/38, 34%, Supplementary Table S5, available at https://doi.org/10.1016/j.esmorw.2024.100075).

### Overall and progression-free survival

OS was calculated from the initiation date of the first treatment for the 286 patients with metastatic disease. Log-rank test statistics showed no significant difference within each cancer entity between patients with *BRAF* class 1 mutations and those with nonclass 1 mutations for colorectal cancer (*P* = 0.99), lung cancer (*P* = 0.93), and melanoma (*P* = 0.35; Figure 2G–I, Supplementary Table S6, available at https://doi.org/10.1016/j.esmorw.2024.100075).

**Figure 3:**
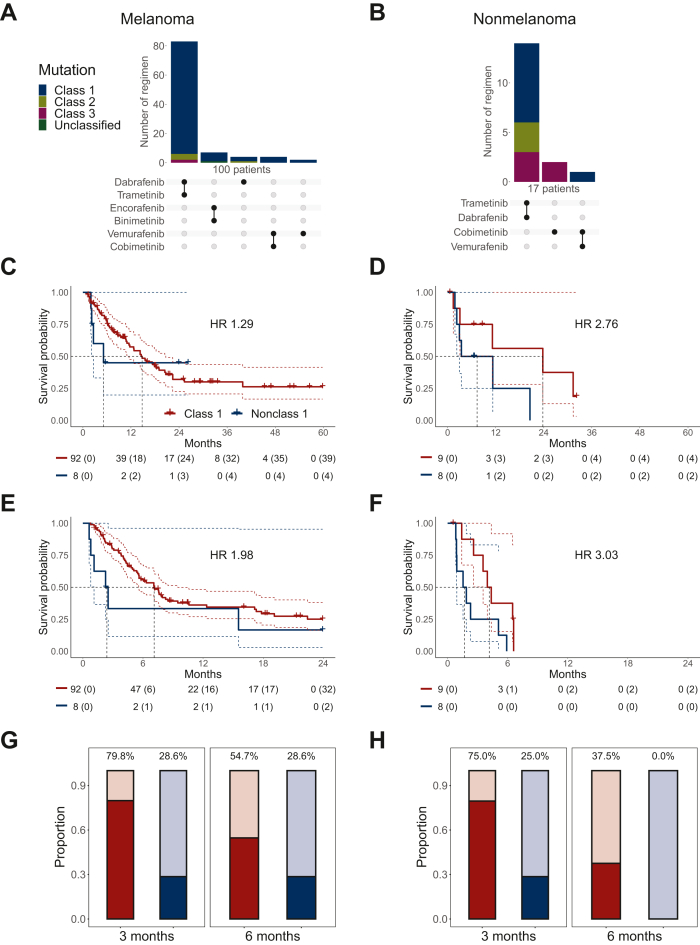
Patients with (A, C, E, G) metastatic melanoma and (B, D, F, H) nonmelanoma treated with BRAF and/or MEK inhibitors. (A, B) UpSet plot of the BRAF/MEK inhibitor regimen administration. (C–F) Kaplan–Meier estimators of (C, D) overall survival and (E, F) progression-free survival. The number of patients at risk at each time point is indicated below the plot, together with the cumulative number of censoring events in parentheses. Survival starts from the first line of treatment for metastatic disease involving a BRAF inhibitor and/or an MEK inhibitor. The censorship, if any, occurs at the last follow-up observed event when the patient is alive. Colored dashed lines represent the upper and lower boundary of the pointwise 95% confidence interval. The median survival is shown with black dashed lines. The number of events and median survival are indicated in Supplementary Table S6, available at https://doi.org/10.1016/j.esmorw.2024.100075. (G, H) Disease control rate at 3, and 6 months. HR, hazard ratio.

We then investigated whether BRAFis or MEKis were effective in nonclass 1 *BRAF*-mutant tumors. The distribution of the different BRAFis and MEKis administered in patients with melanoma and nonmelanoma, with a predominance of dabrafenib/encorafenib in all entities, is shown in Figure 3A and B. Because of the small number of patients treated with BRAFis/MEKis in nonmelanoma disease, we have combined them into one group. Of the 76 nonclass 1 patients with metastatic disease, 16 were treated with MEKis and BRAFis (Supplementary Table S7, available at https://doi.org/10.1016/j.esmorw.2024.100075).

In this group, the start of the first therapy line with BRAFi and/or MEKi was the baseline for assessing overall (OS) and PFS. Patients receiving BRAFi/MEKi in combination with other simultaneous therapies were not considered. Because of the small number of patients in the different subcohorts, log-rank statistics are underpowered and uninformative and were thus waived. However, for OS and PFS, within melanoma and nonmelanoma, nonclass 1 patients tended to have shorter survival (Figure 3C–F). For nonclass 1 melanoma, the median survival was 157 days (OS) and 73 days (PFS). For class 1 melanoma, the median survival was 451 days (OS) and 217 days (PFS, Supplementary Table S6, available at https://doi.org/10.1016/j.esmorw.2024.100075).

The DCR was higher in melanoma class 1 compared with nonclass 1 (Figure 3G) at 3 and 6 months. After 6 months, 54.7% (47/86, 6 were lost to follow-up; Figure 3G) of patients with melanoma class 1 achieved disease control compared with only 28.6% (2/7; 1 was lost to follow-up) nonclass 1 patients. In nonmelanoma, we also observed a better DCR at 3 and 6 months in patients with class 1 mutant disease (Figure 3H).

In nonclass 1 patients, BRAFis/MEKis were given as first- or second-line therapy for metastatic disease (Supplementary Table S7, available at https://doi.org/10.1016/j.esmorw.2024.100075). Many patients who received them as first-line treatment had a short OS (except for one patient with melanoma with a K601E mutation). This may reflect advanced disease at the time of metastatic diagnosis. We then looked at the second-line therapies for nonclass 1 patients. These were single-agent immune checkpoint inhibitors (10 patients), BRAFi/MEKi (*n* = 9), chemotherapy alone (*n* = 6), and immune checkpoint inhibitors + chemotherapy (*n* = 1; Supplementary Tables S7 and S8, available at https://doi.org/10.1016/j.esmorw.2024.100075). We observed no difference in OS, PFS, and DCR (Supplementary Figure S1, available at https://doi.org/10.1016/j.esmorw.2024.100075).

## Discussion

In this observational study, we successfully tested and validated the SPHN infrastructure for harmonizing, combining, and analyzing data from different cancer centers. We investigated the role of the three classes of *BRAF* mutations in cancer.^2^ The most common mutation is the class 1 BRAF p.V600E, which is an established therapeutic target in malignant melanoma and several other entities (e.g. non-small-cell lung cancer, glioma)^28^, ^29^, ^30^ and a tissue-agnostic marker for BRAFis and MEKis.^12^

Non-V600 *BRAF* mutations are much less established as therapeutic targets. Although some therapeutic strategies have been proposed for nonclass 1 *BRAF*-mutated malignancies, such as MEKi monotherapy or combined inhibition of BRAF and MEK for class 2, and a combination with RTK inhibition for class 3 mutations, robust clinical data on targeted therapy are still lacking. There is a small, prospective, nonrandomized, single-institution trial evaluating the combination of binimetinib and encorafenib in patients with cancer with *BRAF* class 2 or 3 mutations. However, the final results have not yet been published (BEAVER trial; NCT03839342). A second small trial investigated MEK inhibition with trametinib monotherapy in nine patients with nonclass 1 *BRAF*-mutated melanoma and found low activity.^31^

Interestingly, we observed lasting responses to dabrafenib and trametinib in two patients with advanced melanoma with class 2 BRAF K601E mutations (15.5 and 26.3 months; Supplementary Table S7, available at https://doi.org/10.1016/j.esmorw.2024.100075). Trametinib may also be effective in this setting, with a duration of response varying from a few months to >36 months.^16^^,^^32^ In this study, we had five patients with K601E-mutant melanomas treated with MEKis and BRAFis. Besides the aforementioned two responders, two had short response durations (34 and 70 days), and one was censored after 70 days (Supplementary Table S6, available at https://doi.org/10.1016/j.esmorw.2024.100075). This suggests that a good proportion of patients with BRAF K601E-mutant melanoma may potentially respond to treatment with BRAFis plus MEKis, making it a reasonable option for patients who progress on immunotherapy. Trametinib can also be effective in other cancer types with K601E mutations, as reported in a patient with NCSLC.^33^

The small sample size of the trials investigating class 2 or 3 *BRAF* mutations highlights the challenge of running clinical trials in such diseases with highly heterogeneous noncanonical mutations. It underlines the importance of capturing information on these patients using novel data-driven approaches. In our cohort, the rate of therapies with targeted agents in *BRAF* class 2 or 3 mutant tumors was 20% (23/116). Most patients received either single-agent MEKis or combinations such as MEKis plus a checkpoint blockade or MEKis plus a BRAFi. This is consistent with the meta-analysis by Dankner et al.,^17^ in which of 238 patients with *BRAF* class 2 and 3 mutations and MAPK-targeted therapy, most (*n* = 87) were treated with MEKi, followed by BRAFi (*n* = 77), BRAFi + MEKi (*n* = 63), and EGFR inhibitor (*n* = 11). Interestingly, in our cohort, the rate of TKI use was the same for class 2 and 3 mutations.

For patients with melanoma, the PFS and the DCR were numerically better with targeted agents against class 1 *BRAF*-mutant disease compared with nonclass 1 mutant disease. No difference was observed at the OS level. These findings are consistent with the analysis by Menzer et al., who showed for rare activating (non-V600E/K) *BRAF* mutations, a better response rate of targeted agents in V600-mutated compared with non-V600 mutated melanomas, with no significant differences in OS and no difference in PFS between the two groups.^34^ The outcomes of nonclass 1 *BRAF* mutant disease were very similar in patients treated in the second or later lines with chemotherapy, immunotherapy, or targeted agents, confirming the impression that current combinations of targeted agents are, at best, moderately active against *BRAF* class 2 or 3 mutant disease. As nonclass 1 *BRAF* mutant tumors are relatively common overall, accounting for roughly one-third to one-fourth of *BRAF*-mutant diseases, the exploration of new avenues, such as next-generation BRAFis that specifically target these mutations, would be very welcome.

As a limitation, due to the retrospective and organizational nature of this study, our analysis may be biased by the tendency to test less advanced diseases with hotspot gene panel assays, which may miss rare, lesser-known mutations, potentially leading to a biased ratio of canonical to rare *BRAF* mutations. In addition, treatment rates may not be complete, as some patients may have received additional lines of treatment outside the five university hospitals later in the course of their disease.

In conclusion, besides successfully demonstrating the usefulness of the innovative SPO bioinformatics infrastructure for precision oncology challenges, our study showed that patients with nonclass 1 *BRAF*-mutant disease received overall fewer targeted agents and demonstrated a trend to reduced PFS and DCR outcomes following BRAFi/MEKi therapy in second or later lines. This shows that existing precision oncology strategies inadequately address cancers with nonclass 1 mutations. However, a few specific class 2 mutations, such as K601E, may be responsive to approved BRAFis and MEKis, suggesting that BRAF-mutant disease could benefit from a mutation-specific rather than class-specific treatment approach. These findings further underscore the urgent need for new treatment strategies against nonclass 1 *BRAF*-mutated cancers and advocate the careful consideration of *BRAF* mutations in treatment allocation across cancer types.
