# Supplementary material for: Real-world occurrence, therapy, and outcome of patients with class 2 or 3 BRAF compared with class 1 BRAF-mutated cancers
Source: ESMO Real World Data Digit Oncol. 2024 Sep 25;6:100075. doi: 10.1016/j.esmorw.2024.100075 (PMC12836636; doi:10.1016/j.esmorw.2024.100075)
Supplement: Supplementary Material [file mmc1.docx]

Supplementary Material

## Supplementary Figures

##
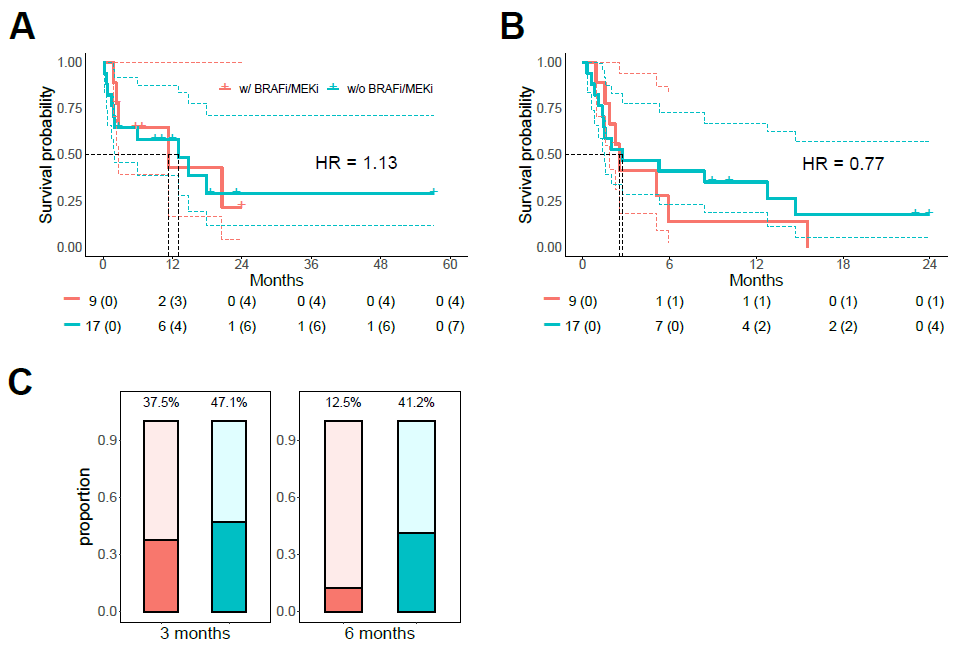


## Supplementary Figure 1. Kaplan-Meier estimators of: overall survival of non-class 1 patients from the second line (A); progression-free survival of non-class 1 patients from the second line (B). Clinical benefit rates at 8, 14 and 20 weeks are indicated for non-class 1 patients from the second line of antineoplastic systemic therapy (C). The number of patients at risk at each time point is indicated below the plot, together with the cumulative number of censoring events in parentheses. The *P*-values indicated in the figure are computed from the log-rank test statistic. Survival starts from the first metastatic line involving a BRAF inhibitor and/or a MEK inhibitor. The censorship, if any, occurs in the last follow-up observed event when the patient is alive. Dashed lines represent the upper and lower boundary of the pointwise 95% confidence interval. Median survival is shown with black dashed lines. HR: hazard ratio.

## Supplementary Tables

**Supplementary Table 1. Classification of BRAF mutations observed in the cohort.**

| **BRAF mutation** | **Class** | **Reference** |
| --- | --- | --- |
| V600D | Class 1 | Yao et al., Nature 2017 [pmid 28783719] |
| V600E | Class 1 | Yao et al., Nature 2017 [pmid 28783719] |
| V600K | Class 1 | Yao et al., Nature 2017 [pmid 28783719] |
| V600R | Class 1 | Yao et al., Nature 2017 [pmid 28783719] |
| E586K | Class 2 | Dankner et al., Oncogene 2018 [pmid 29540830] |
| F468C | Class 2 | Krebs et al., Sci Rep. 2022 [pmid 35869122] |
| F595V | Class 2 | Krebs et al., Sci Rep. 2022 [pmid 35869122] |
| G464E | Class 2 | Yao et al., Nature 2017 [pmid 28783719] |
| G464R | Class 2 | Dankner et al., Oncogene 2018 [pmid 29540830] |
| G464V | Class 2 | Yao et al., Nature 2017 [pmid 28783719] |
| G469A | Class 2 | Yao et al., Nature 2017 [pmid 28783719] |
| G469I | Class 2 | Krebs et al., Sci Rep. 2022 [pmid 35869122] |
| G469R | Class 2 | Yao et al., Nature 2017 [pmid 28783719] |
| G469V | Class 2 | Yao et al., Nature 2017 [pmid 28783719] |
| I582V | Class 2 | Krebs et al., Sci Rep. 2022 [pmid 35869122] |
| K483Q | Class 2 | Krebs et al., Sci Rep. 2022 [pmid 35869122] |
| K601E | Class 2 | Yao et al., Nature 2017 [pmid 28783719] |
| K601N | Class 2 | Yao et al., Nature 2017 [pmid 28783719] |
| L485F | Class 2 | Lin et al., Journal of Translational Medicine 2019 [pmid 31470866] |
| L597Q | Class 2 | Yao et al., Nature 2017 [pmid 28783719] |
| L597R | Class 2 | Dankner et al., Oncogene 2018 [pmid 29540830] |
| L597S | Class 2 | Dankner et al., Oncogene 2018 [pmid 29540830] |
| N581H | Class 2 | Krebs et al., Sci Rep. 2022 [pmid 35869122] |
| T470R | Class 2 | Krebs et al., Sci Rep. 2022 [pmid 35869122] |
| T599I | Class 2 | Dankner et al., Oncogene 2018 [pmid 29540830] |
| D594E | Class 3 | Dankner et al., Oncogene 2018 [pmid 29540830] |
| D594G | Class 3 | Yao et al., Nature 2017 [pmid 28783719] |
| D594N | Class 3 | Yao et al., Nature 2017 [pmid 28783719] |
| D594V | Class 3 | Dankner et al., Oncogene 2018 [pmid 29540830] |
| F595L | Class 3 | Yao et al., Nature 2017 [pmid 28783719] |
| G466A | Class 3 | Yao et al., Nature 2017 [pmid 28783719] |
| G466E | Class 3 | Yao et al., Nature 2017 [pmid 28783719] |
| G466R | Class 3 | Dankner et al., Oncogene 2018 [pmid 29540830] |
| G466V | Class 3 | Yao et al., Nature 2017 [pmid 28783719] |
| G469E | Class 3 | Yao et al., Nature 2017 [pmid 28783719] |
| G596C | Class 3 | Dankner et al., Oncogene 2018 [pmid 29540830] |
| G596D | Class 3 | Yao et al., Nature 2017 [pmid 28783719] |
| G596R | Class 3 | Yao et al., Nature 2017 [pmid 28783719] |
| N581I | Class 3 | Yao et al., Nature 2017 [pmid 28783719] |
| N581S | Class 3 | Yao et al., Nature 2017 [pmid 28783719] |
| R603Q | Class 3 | Krebs et al., Sci Rep. 2022 [pmid 35869122] |
| S467L | Class 3 | Yao et al., Nature 2017 [pmid 28783719] |
| S467L | Class 3 | Dankner et al., Oncogene 2018 [pmid 29540830] |
| D449V | Unclassified | Krebs et al., Sci Rep. 2022 [pmid 35869122] |
| E586K | Unclassified | Lokhandwala et al., BCM Cancer 2019 [pmid 31277584] |
| F707Y | Unclassified | Krebs et al., Sci Rep. 2022 [pmid 35869122] |
| G606E | Unclassified | Krebs et al., Sci Rep. 2022 [pmid 35869122] |
| I659M | Unclassified | Lin et al., Journal of Translational Medicine 2019 [pmid 31470866] |
| L441F | Unclassified | Krebs et al., Sci Rep. 2022 [pmid 35869122] |
| L584F | Unclassified | Krebs et al., Sci Rep. 2022 [pmid 35869122] |
| R239Q | Unclassified | Krebs et al., Sci Rep. 2022 [pmid 35869122] |
| S605G | Unclassified | Krebs et al., Sci Rep. 2022 [pmid 35869122] |
| S607F | Unclassified | Lokhandwala et al., BCM Cancer 2019 [pmid 31277584] |
| T440I | Unclassified | Krebs et al., Sci Rep. 2022 [pmid 35869122] |
| T491S | Unclassified | Krebs et al., Sci Rep. 2022 [pmid 35869122] |
| E13K | Unclassified | Not in kinase domain |
| G392R | Unclassified | Not in kinase domain |
| G466fs | Unclassified | Not SNV |
| K150Sfs*32 | Unclassified | Not SNV |
| M620I | Unclassified | Krebs et al., Sci Rep. 2022 [pmid 35869122] |
| N486_P490del | Unclassified | Not SNV |
| P321L | Unclassified | Not in kinase domain |
| R146W | Unclassified | Not in kinase domain |
| R424Q | Unclassified | Not in kinase domain |
| R719C | Unclassified | Not in kinase domain |
| S363F | Unclassified | Not in kinase domain |
| T599dup | Unclassified | Not SNV |

**Supplementary Table 2**. Patients with multiple BRAF mutations.

| **Patient** | **HGVSp (NP_004324.2)** | **BRAF mutation class** | **VAF [%]** | **Patient classification** |
| --- | --- | --- | --- | --- |
| 1 | p.Glu586Lys | Unclassified | 8.5 | Class 2 |
|  | p.Gly469Ala | Class 2 | 29 |  |
| 2 | p.Arg603Gln | Class 3 | 23 | Ambiguous |
|  | p.Val600Glu | Class 1 | 49 |  |
| 3 | p.Ser467Leu | Class 3 | 33 | Class 3 |
|  | p.Thr440Ile | Unclassified | 5.8 |  |
| 4 | p.Ser607Phe | Unclassified | 47 | Ambiguous |
|  | p.Val600Asp | Class 1 | 46 |  |
| 5 | p.Lys601Glu | Class 2 | 66 | Unclassified non-class 1 |
|  | p.Ser363Phe | Unclassified | 66 |  |
| 6 | p.Phe707Tyr | Unclassified | 0.11 | Class 1 |
|  | p.Val600Glu | Class 1 | 45 |  |
| 7 | p.Ser605Gly | Unclassified | 46 | Ambiguous |
|  | p.Val600Glu | Class 1 | 46 |  |
| 8 | p.Val600Lys | Class 1 | 54 | Class 1 |
|  | p.Asp449Val | Unclassified | 14 |  |
| 9 | p.Gly469Ala | Class 2 | NA | Unclassified non-class 1 |
|  | p.Thr470Arg | Unclassified | NA |  |

**Supplementary Table 3**. Administrative sex per diagnosis for patients with BRAF mutations. The Binomial exact test’s confidence intervals (CI) are represented.

| **Diagnosis** | **# female** | **# male** | **Proportion of male [95% CI]** | **Binomial test *P*val** (H0 = 50%) |
| --- | --- | --- | --- | --- |
| colorectal | 36 | 40 | 52.6% [40.8% – 64.2%] | 0.731 |
| lung | 47 | 49 | 51.0% [40.6% – 61.4%] | 0.919 |
| melanoma | 118 | 179 | 60.3% [54.5% – 65.9%] | 0.000478 |
| other | 42 | 35 | 45.5% [34.1% – 57.2%] | 0.494 |

**Supplementary Table 4**. Male / Female ratios in the different BRAF mutation classes stratified by disease entity. Fisher exact test H0: ratios are the same in each class.

| **Diagnosis** | **Class 1** | **Class 2** | **Class 3** | **Unclassified** | **Fisher test *P*val** |
| --- | --- | --- | --- | --- | --- |
| colorectal | 32 / 29 | 1 / 1 | 5 / 5 | 2 / 1 | 1 |
| lung | 15 / 18 | 14 / 16 | 17 / 11 | 3 / 2 | 0.635 |
| melanoma | 152 / 103 | 19 / 6 | 6 / 7 | 2 / 2 | 0.235 |
| other | 16 / 27 | 6 / 7 | 6 / 5 | 7 / 3 | 0.275 |

**Supplementary Table 5**. Number of patients for each type of systemic drug treatment in any therapeutic setting (384 patients).

| **Diagnosis** | **Treatment type** | | **BRAF mut. class 1** | **BRAF mut. class 2** | **BRAF mut. class 3** | **BRAF mut. unclassified** |
| --- | --- | --- | --- | --- | --- | --- |
| Colorectal cancer | Chemo | | 27 | 1 | 5 | 2 |
|  | Immuno | Anti-CTLA4 | 3 | 0 | 0 | 0 |
|  |  | Anti-PD1 | 5 | 0 | 0 | 0 |
|  |  | ***Any Immuno*** | **5** | **0** | **0** | **0** |
|  | Targeted | Anti-EGFR | 10 | 1 | 1 | 1 |
|  |  | Anti-VEGFR | 12 | 0 | 3 | 1 |
|  |  | BRAFi | 3 | 0 | 0 | 0 |
|  |  | MEKi | 1 | 0 | 1 | 0 |
|  |  | TKi | 2 | 0 | 1 | 0 |
|  |  | ***Any Targeted*** | **17** | **1** | **3** | **1** |
| NSCLC/  SCLC | Chemo | | 14 | 16 | 18 | 4 |
|  | Immuno | Anti-CTLA4 | 0 | 1 | 2 | 0 |
|  |  | Anti-PD1 | 8 | 6 | 10 | 1 |
|  |  | Anti-PDL1 | 1 | 1 | 4 | 1 |
|  |  | ***Any Immuno*** | **9** | **7** | **12** | **2** |
|  | Targeted | Anti-VEGFR | 0 | 0 | 1 | 0 |
|  |  | BRAFi | 7 | 3 | 3 | 0 |
|  |  | MEKi | 7 | 3 | 3 | 0 |
|  |  | TKi | 1 | 0 | 1 | 0 |
|  |  | ***Any Targeted*** | **8** | **3** | **5** | **0** |
| Melanoma | Chemo | | 18 | 0 | 0 | 0 |
|  | Immuno | Anti-CTLA4 | 100 | 7 | 2 | 1 |
|  |  | Anti-PD1 | 176 | 16 | 5 | 3 |
|  |  | Recombinant cytokine | 6 | 0 | 0 | 0 |
|  |  | Oncolytic virus | 6 | 0 | 1 | 0 |
|  |  | ***Any Immuno*** | **186** | **17** | **6** | **3** |
|  | Targeted | Anti-CD20 | 1 | 0 | 0 | 0 |
|  |  | Anti-VEGFR | 1 | 0 | 0 | 0 |
|  |  | BRAFi | 129 | 5 | 2 | 1 |
|  |  | MEKi | 126 | 4 | 2 | 1 |
|  |  | TKi | 3 | 0 | 0 | 0 |
|  |  | ***Any Targeted*** | **131** | **5** | **2** | **1** |
| Other | Chemo | | 17 | 3 | 6 | 6 |
|  | Immuno | Anti-CTLA4 | 3 | 0 | 1 | 0 |
|  |  | Anti-PD1 | 8 | 1 | 1 | 0 |
|  |  | Anti-PDL1 | 2 | 0 | 0 | 1 |
|  |  | ***Any Immuno*** | **10** | **1** | **1** | **1** |
|  | Targeted | AB-drug conjugate | 1 | 0 | 0 | 1 |
|  |  | Anti-CD20 | 2 | 0 | 0 | 1 |
|  |  | Anti-HER2 | 0 | 1 | 0 | 0 |
|  |  | Anti-VEGFR | 1 | 1 | 2 | 0 |
|  |  | BRAFi | 5 | 0 | 1 | 0 |
|  |  | MEKi | 3 | 0 | 2 | 0 |
|  |  | PARPi | 0 | 0 | 0 | 1 |
|  |  | TKi | 2 | 0 | 0 | 1 |
|  |  | ***Any Targeted*** | **10** | **1** | **3** | **2** |

**Supplementary Table 6**. Overall survival (OS) and progression-free survival (PFS) for patients with class 1 and non-class 1 BRAF mutations. Corresponding figures are indicated. CI = confidence interval; HR = hazard ratio; NR = not reached.

| **Figure panel** | **Survival** | **Cohort** | **Arm** | **# Events / total N** | **median survival in days [95% CI]** | **median follow-up in days [95% CI]** | **HR [95% CI]** |
| --- | --- | --- | --- | --- | --- | --- | --- |
| 2G | OS | Colorectal cancer | Class 1 | 23/13 | 675 [390 - NR] | 1096 [487 - NR] | reference |
| 2G | OS | Colorectal cancer | Non-class 1 | 7/4 | 1044  [297 - NR] | 1514 [423 - NR] | 1.01 [0.329 - 3.12] |
| 2H | OS | Lung cancer | Class 1 | 16/11 | 719 [169 - NR] | 1309 [982 - NR] | reference |
| 2H | OS | Lung cancer | Non-class 1 | 33/18 | 581 [316 - NR] | 834 [458 - NR] | 0.964 [0.451 - 2.06] |
| 2I | OS | Melanoma | Class 1 | 161/67 | 1217 [688 - NR] | 992 [850 - 1123] | reference |
| 2I | OS | Melanoma | Non-class 1 | 23/10 | NR [192 - NR] | 874 [464 - NR] | 1.37 [0.704 - 2.66] |
| 3C | OS | Melanoma BRAFi/MEKi | Class 1 | 92/53 | 451 [351 - 631] | 887 [650 - 1383] | reference |
| 3C | OS | Melanoma BRAFi/MEKi | Non-class 1 | 8/4 | 157 [80 - NR] | 730 [171 - NR] | 1.29 [0.464 - 3.58] |
| 3D | OS | Non-melanoma BRAFi/MEKi | Class 1 | 9/5 | 725 [341 - NR] | 982 [200 - NR] | reference |
| 3D | OS | Non-melanoma BRAFi/MEKi | Non-class 1 | 8/6 | 224 [90 - NR] | 204 [199 - NR] | 2.76 [0.362 - 11.3] |
| 3E | PFS | Melanoma BRAFi/MEKi | Class 1 | 92/60 | 217 [170 - NR] | 662 [490 - NR] | reference |
| 3E | PFS | Melanoma BRAFi/MEKi | Non-class 1 | 8/6 | 73 [34 - NR] | 730 [70 - NR] | 1.98 [0.851 - 4.60] |
| 3F | PFS | Non-melanoma BRAFi/MEKi | Class 1 | 9/7 | 127 [108 - NR] | 200 [200 - NR] | reference |
| 3F | PFS | Non-melanoma BRAFi/MEKi | Non-class 1 | 8/8 | 52 [28 - NR] | NR [NR - NR] | 3.03 [0.973 - 9.45] |

**Supplementary Table 7.** Characteristics of non-class 1 patients treated with MEK and BRAF inhibitors. Censoring is indicated with a (+). Overall survival and progression free survival since start of BRAFi/MEKi treatment are indicated.

|  | **Sex** | **Age at diag.** | **Diagnostic** | **BRAF mutation** | **BRAFi / MEKi** | **Metastatic Line** | **Overall survival (days)** | **Progression free survival (days)** | **Best objective response** |
| --- | --- | --- | --- | --- | --- | --- | --- | --- | --- |
| 1 | M | 86 | C43: melanoma | D594N (cl. 3) | Dabrafenib, Trametinib | 1 | 61 | 24 | PD |
| 2 | M | 71 | C43: melanoma | K601E (cl. 2) | Dabrafenib, Trametinib | 2 | 80 | 69 | PD |
| 3 | M | 83 | C34: NSCLC | G596R (cl. 3) | Dabrafenib, Trametinib | 1 | 199 (+) | 70 | PD |
| 4 | F | 71 | C34: NSCLC | G464V (cl. 2) | Dabrafenib, Trametinib | 1 | 90 | 27 | PD |
| 5 | M | 81 | C34: NSCLC | E586K (uncl.),  G469A(cl.2) | Dabrafenib, Trametinib | 1 | 105 | 25 | PD |
| 6 | M | 81 | C43: melanoma | L597Q (cl. 2) | Dabrafenib | 1 | 67 | 19 | PD |
| 7 | F | 41 | C56: ovarian cancer | G469E (cl. 3) | Dabrafenib, Trametinib | 2 | 627 | 155 | PR |
| 8 | M | 71 | C34: NSCLC | N581I (cl. 3) | Dabrafenib, Trametinib | 2 | 344 | 180 | SD |
| 9 | M | 66 | C31: Accessory sinuses cancer | N581S (cl. 3) | Cobimetinib | 2 | 56 | 28 | PD |
| 10 | M | 44 | C19: colorectal cancer | D594G (cl. 3) | Cobimetinib | 2 | 70 | 57 | PD |
| 11 | F | 38 | C43: melanoma | K601E (cl. 2) | Dabrafenib, Trametinib | 1 | 157 | 34 | PD |
| 12 | M | 74 | C43: melanoma | S467L (cl. 3) | Dabrafenib, Trametinib | 2 | 171 (+) | 77 | PD |
| 13 | M | 76 | C43: melanoma | K601E (cl. 2) | Dabrafenib, Trametinib | 2 | 730 (+) | 474 | CR |
| 14 | F | 54 | C34: NSCLC | G469V (cl. 2) | Dabrafenib, Trametinib | 2 | 204 (+) | 47 | PD |
| 15 | F | 74 | C43: melanoma | K601E (cl. 2), S363F (uncl.) | Binimetinib, Encorafenib | 2 | 70 (+) | 70 (+) | PR |
| 16 | M | 75 | C43: melanoma | K601E (cl. 2) | Dabrafenib, Trametinib | 1 | 801 (+) | 801 (+) | CR |

**Supplementary Table 8.** Characteristics of second line non-class 1 patients not treated with MEK and BRAF inhibitors. Censoring is indicated with a (+). Overall survival since start of the second line treatment is indicated.

|  | **Sex** | **Age at diag.** | **Diagnostic** | **BRAF mutation** | **Second line drugs** | **Overall survival (days)** | **Progression free survival (days)** |
| --- | --- | --- | --- | --- | --- | --- | --- |
| 1 | F | 66 | C34: lung | G469A (cl. 2) | Ipilimumab, Nivolumab | 61 | 61 |
| 2 | F | 78 | C34: lung | D594G (cl. 3) | Ipilimumab, Nivolumab | 47 | 47 |
| 3 | M | 78 | C34: lung | N581S (cl. 3) | Pemetrexed, Bleomycin, Carboplatin | 545 | 256 |
| 4 | M | 83 | C34: lung | G596R (cl. 3) | Ipilimumab, Nivolumab, Carboplatin, Pemetrexed | 80 (+) | 41 |
| 5 | F | 56 | C54: corpus uteri | F595V (cl. 2) | Nivolumab | 308 (+) | 308 (+) |
| 6 | M | 75 | C24: biliary tract | D594G (cl. 3) | Oxaliplatin, Fluorouracil | 183 | 161 |
| 7 | M | 57 | C34: lung | G596R (cl. 3) | Nivolumab | 1741(+) | 1741(+) |
| 8 | F | 70 | C34: lung | G596R (cl. 3) | Nivolumab | 364 (+) | 83 |
| 9 | M | 50 | C34: lung | G469A (cl. 2) | Cisplatin, Vinorelbine | 272 (+) | 272 (+) |
| 10 | F | 51 | C34: lung | D594N (cl. 3) | Durvalumab | 701 (+) | 701 (+) |
| 11 | F | 62 | C19: colorectal | S467L (cl. 3), T440I (unlc.) | Trifluridine and Tipiracil | 564(+) | 389 |
| 12 | F | 35 | C50: breast | E13K (uncl.) | Atezolizumab | 56 | 34 |
| 13 | M | 63 | C34: lung | D594G (cl. 3) | Docetaxel | 18 | 18 |
| 14 | F | 38 | C43: melanoma | K601E (cl. 2) | Nivolumab | 9 | 9 |
| 15 | F | 68 | C34: lung | G466V (cl. 3) | Nivolumab | 399 | 45 |
| 16 | F | 81 | C18: colorectal | G469E (cl. 3) | Capecitabine | 25 | 25 |
| 17 | F | 85 | C34: lung | K601E (cl. 2) | Durvalumab | 449 | 449 |

**Supplementary Methods:** Genomic profiling was performed in each university hospital as follows:

Geneva:

- - - - Ion AmpliSeq Cancer Hotspot Panel v2, Ion Proton sequencer
      - Ion AmpliSeq Comprehensive Cancer Panel, Ion Proton sequencer
      - Custom 100-gene “hotspot” panel (Agilent SureSelect XT HS), Illumina NextSeq 500/550 sequencer
      - Custom 400-gene panel (Agilent SureSelect XT HS), Illumina NextSeq 500/550 sequencer

Lausanne:

- - - - NextSeqTM 550 System platform (Illumina) using the xGen® Lockdown® IDT Large Custom Cancer Panel IPA, covering the complete coding sequences of approximately 400 genes (original panel developed in-house, list available on request)
      - NextSeqTM 550 System platform (Illumina) using the Twist Large Custom Cancer Panel IPA (Twist Bioscience), covering the complete coding sequences of approximately 400 genes (original panel developed in-house, list available on request)
      - Ion GeneStudio S5 System platform (Ion Torrent, Thermo Fisher Scientific) using the Ion AmpliSeqTM Custom Cancer Hotspot Panel IPA (custom version) covering 218 hotspot regions of 52 genes.
      - Ion Personal Genome Machine (PGMTM, Ion Torrent, Thermo Fisher Scientific) using the Ion AmpliSeqTM Custom Cancer Hotspot Panel IPA (custom version), covering 218 hotspot regions of 52 genes.

Zurich:

- - - - FoundationOne®CDx on Illumina platform
      - Oncomine panels on Ion torrent platform

Basel:

- - - - NGS Comprehensive
      - Oncomine Solid
      - Oncomine Focus
      - Oncomine TMB+Comprehensive V3 DNA+RNA
      - Oncomine Solid+ev.Fusion
      - Oncomine Focus DNA
      - Oncomine Lung cfDNA
      - Comprehensive V3 DNA+RNA
      - Oncomine Solid+Fusion
      - Oncomine Solid+Sanger
      - Oncomine Solid, possibly Fusion
      - Comprehensive V3 DNA
      - Comprehensive V3 DNA+TMB
      - CHPv2
      - CHPv2 + Pyrosequenzierung
      - CHPv2 + Sanger
      - CHPV2
      - CHPv2/Sanger
      - ddPCR
      - Sanger
      - Custom Lymphoma

Bern:

- - - - Ion AmpliSeq Cancer Hotspot Panel
      - Ion AmpliSeq Cancer Hotspot Panel v2
      - Ion AmpliSeq Oncomine Comprehensive Cancer Panel
      - Ion AmpliSeq Oncomine Comprehensive Fusion Panel
      - Ion AmpliSeq Oncomine Comprehensive Panel
      - Ion AmpliSeq Oncomine Focus Cancer Panel
      - Ion AmpliSeq Oncomine Focus Fusion Panel
      - Ion AmpliSeq Oncomine Solid Colon & Lung Cancer Panel
      - Ion AmpliSeqTM Hotspot Cancer Panel, Ion Torrent PGM
      - Pyrosequencing BRAF Exon 15
